# Supplementary material for: Identification of photoperiod-induced specific miRNAs in the adrenal glands of Sunite sheep (Ovis aries)
Source: Front Vet Sci. 2022 Jul 22;9:888207. doi: 10.3389/fvets.2022.888207 (PMC9354845; doi:10.3389/fvets.2022.888207)
Supplement: Supplementary file 1 [file Data_Sheet_1.ZIP › Supplementary file/miRDeep2 file.zip!/miRDeep2 file/Know_Structure/LP42A1/oar-mir-329b.pdf]

miRBase precursor : oar-mir-329b
Total read count : 1318
oar-miR-329b-5p read count : 15
oar-miR-329b-3p read count : 1298
remaining reads : 5

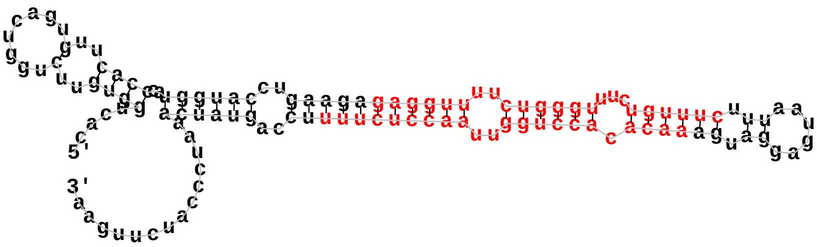

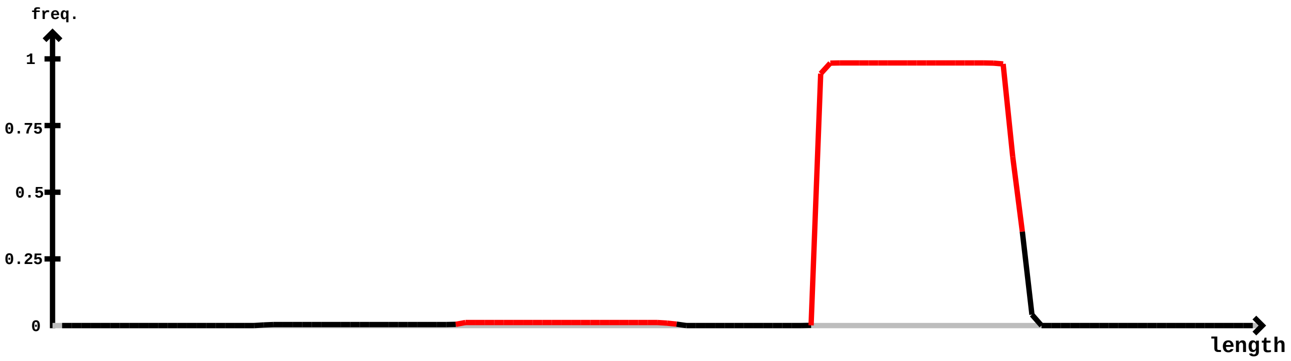


```
                                                                               oar-miR-329b-3p
                                         oar-miR-329b-5p
5'- cacugguguucuggucaguguucacccaugguaccugaagagagguuuucuggguuucuguuucuuuaaugaggaugaaacacaccugguuaaccucuuuuccaguaucaaaucccaucuugaa  -3'  exp
    ....((((..(........)..))))..((((((..((((((((((..((((((...((((((.((......)).)))))).))))))..))))))))))..))))))................   reads  mm
    ....................uucacccaugguaccugaag....................................................................................   1      0
    ....................uucacccaugguaccugaaga...................................................................................   2      0
    .....................ucacccaugguaccugaaga...................................................................................   2      0
    ........................................agagguuuucuggguuucuguu..............................................................   1      0
    ........................................agagguuuucuggguuucuguuuc............................................................   1      0
    .........................................gagguuuucuggguuucuguu..............................................................   2      0
    .........................................gagguuuucuggguuucuguuu.............................................................   5      0
    .........................................gagguuuucuggguuucuguuuc............................................................   6      0
    .............................................................................aaacacaccugguuaaccucu..........................   1      0
    ..............................................................................aacacaccugguuaaccu............................   1      0
    ..............................................................................aacacaccugguuaaccuc...........................   4      0
    ..............................................................................aacacaccugguuaaccucu..........................   454    0
    ..............................................................................aacacaccugguuaaccucuu.........................   369    0
    ..............................................................................aacacaccugguuaaccucuuu........................   395    0
    ..............................................................................aacacaccugguuaaccucuuuu.......................   21     0
    ...............................................................................acacaccugguuaaccucu..........................   3      0
    ...............................................................................acacaccugguuaaccucuu.........................   1      0
    ...............................................................................acacaccugguuaaccucuuu........................   15     0
    ...............................................................................acacaccugguuaaccucuuuu.......................   33     0
    ................................................................................cacaccugguuaaccucu..........................   1      0
```
